# Beyond multiport DaVinci®: a closer look at less commonly used robotic systems in resectional colorectal surgery

**DOI:** 10.1007/s11701-026-03329-1

**Published:** 2026-04-07

**Authors:** Rahul Bhome, Subash P Vasudevan

**Affiliations:** 1 Colorectal Unit, Colchester Hospital, East Suffolk and North Essex Foundation Trust, Turner Rd, Colchester CO4 5JL UK; 2 University Surgery, University of Southampton, Level C South Academic Block, Southampton General Hospital; 3 Anglia Ruskin University, East Rd, Cambridge CB1 1PT

**Keywords:** Robotic, colorectal, cancer, inflammatory bowel disease, diverticular, Versius, Senhance, HUGO, Dexter, DaVinci SP

## Abstract

Colorectal surgery has seen an exponential rise in use of robotics in the past decade. Whilst Intuitive’s multiport platforms dominate the market share, several other systems are in use across the globe. This review sets out to describe the components of these systems, discuss their pros and cons, and analyze the data which underpins their safety, efficacy and efficiency in resectional colorectal surgery.

## 1 Introduction

**Figure 1:**
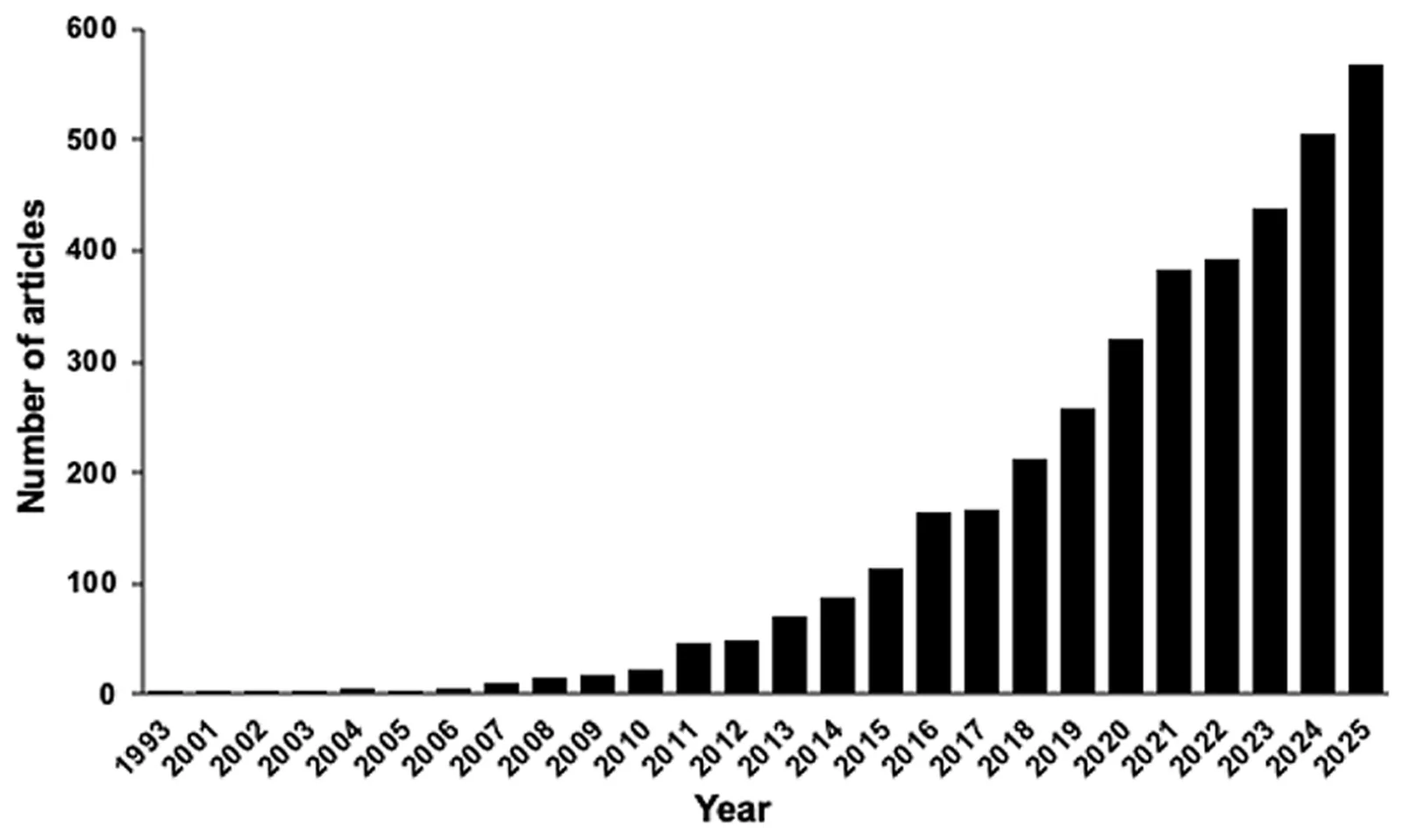
The publication of studies related to robotic colorectal surgery has risen exponentially. Graphical representation of articles indexed by PubMed over time, containing the search terms **(a)** “robotics” and **(b)** “colorectal surgery”.

There has been an exponential rise in the use of robotic systems in colorectal surgery over the past twenty years (Figure 1), fueled by technological advancement, greater awareness of the potential benefits for patients and surgeons and a growing evidence base to confirm its feasibility, safety and advantage over existing techniques [1–3].

The benefit of robotic surgery reflects the 3D optics, wristed instruments and tremor filtration, which allow for more precise technique over existing laparoscopic platforms, with studies suggesting greater accuracy and reduced tissue trauma [4]. With the greatest investment in research and development, measured in terms of time and financial cost, closely patented technology and a successful marketing strategy, Intuitive Surgical has greater than 70% market share in this sector, having sold over 10 000 systems worldwide, with reported use in over 3 million procedures in 2025 alone. Unsurprisingly, the largest trials of robotics in colorectal surgery to date have employed the DaVinci system [1–3, 5].

However, with several of Intuitive’s early patents having expired, several alternative robotic systems have emerged in the past decade for use in abdominal surgery. Like the DaVinci multiport platforms, these are teleoperated master-slave systems with limited autonomy [6]. Broadly, the systems can be grouped by console type (open or closed) and whether the arms operate from a single fixed patient cart or multiple modular arm carts (modular system).

DaVinci’s closed console set-up has been criticized for limiting communication between primary surgeon and other members of the team (bedside assistant, scrub nurse, anesthetist, trainee surgeon) and the fact that the surgeon is not immediately able to operate at the bedside without scrubbing up [7, 8]. This has led to the development of several open console systems, some of which are discussed below. These systems are typically used in a single console set up. Similarly, DaVinci’s large single pivot tower makes it less portable than alternative systems and makes it more challenging to store. Developers have sought to exploit these limitations in novel systems.

This review seeks to document the alternative systems that are being used in colorectal surgery across the globe, as a reference for surgeons, the wider theatre team and patients. Here, we describe these systems in more detail, their differences, pros and cons and most importantly, the outcome data which reflects their real-world use in colorectal surgery.

## 2 Robotic systems

### 2.1 Versius®

The Versius system, designed by Cambridge Medical Robotics in the UK, received European conformity mark approval in 2019 and ‘de novo’ FDA approval in 2024 (for cholecystectomy). Unverified reports suggest it has been used in 40 000 procedures to date across the world. Versius has an open surgeon console and up to five modular patient carts. Hand controls are pistol grip in design and there are no food pedals so that endoscope control, clutching and instrument switching are all hand-controlled. Instruments are wristed with seven degrees of freedom, with a fenestrated grasper, dissector, monopolar hook, needle driver and 30^0^ telescope on offer. However, it lacks an integrated energy device or stapler, which are well established in Intuitive’s multiport systems. However, the Versius Plus system, which got ‘501k’ FDA approval in 2025, is an upgraded iteration with compatible ultrasonic shears and near infrared imaging capability.

The first report of Versius use in colorectal surgery was published in 2020, when the system was used at a UK NHS ‘cold’ site during the Covid pandemic, showing that robotic surgery was feasible during this time [9]. Two patients had left-sided colonic resections for colorectal cancer, with no major complications (Clavien-Dindo >2) or mortality.

The first Versius series from Scotland was published in 2021, including 32 selected low-risk patients (ASA 1-2; T1-3 tumors; no ultra-low rectal tumors) who had colorectal cancer resections at a single center [10]. Median operative time was 221 min (111 min console time) for right sided and 319 min (204 min console time) for left sided resections. All resections were pathologically R0. There were no open conversions. Median length of stay (LOS) was four days and there were no major complications.

The largest UK series was published in 2021, with a prospective cohort of 68 patients having resectional (cancer, inflammatory bowel disease (IBD), diverticular) or restorative surgery (Hartmann’s reversal, ileocolonic hook up) at a single center [11]. Ninety-six percent of patients had resection and of these, 83% had cancer resection. Median docking time was 11 min and console time 159 min. The open conversion rate was 4%, LOS 6 days and major complication rate 7%. This series incorporates the initial 23 patients that were described a year earlier [12].

In 2022, Puntambekar et al. reported their series from a single Indian center of 31 patients having rectal cancer resection by a hybrid laparoscopic/ Versius approach [13]. Versius was used solely for the total mesorectal excision (TME), with a traditional laparoscopic approach for the abdominal phase of the procedure. Median console time was 51 min for the TME. LOS was 7 days on average. All specimens were R0. One patient had a major complication.

As part of a retrospective report of 175 patients having robotic surgery using Versius from a single German center, 11 patients had right hemicolectomy and 13 had low anterior resection (LAR) [14]. All patients had R0 resections. Median operative time was 178 and 214 min respectively. There were no open conversions. Median LOS was 10 and 9 days respectively. One patient had a major complication.

The first Italian cohort of six patients (three right sided and three left sided colorectal cancer resections) was reported in 2022 [15]. Median operative time was 160 min for right sided and 250 min for left sided resections. There were no open conversions. There were no major complications.

Data from the prospective Versius registry was reported in 2023, summarizing the studies described above [16]. Three hundred and seventy-four patients had colorectal procedures recorded between 2019 and 2022, of which 162 had anterior resection. Median operative time was 260 min. The rate of open conversion was 7% overall. LOS was 9 days on average. Five percent of patients had major complications. The 30-day readmission rate was 4% and 90-day mortality rate was 0.3%.

Looking specifically at surgeon-outcomes rather than patient outcomes, the VOLCANO trial randomized 60 colorectal resections to a Versius or traditional laparoscopic approach (2:1) and compared ergonomics and cognitive strain in the operators [17]. Rapid Entire Body Assessment (REBA) posture analysis by objective assessment of sequential surgeon photographs was significantly less in the Versius group, reducing the injury risk category from ‘medium’ to ‘low’. Cognitive strain was also significantly less in the Versius arm, which may be due to fact that Versius does not have foot pedals, with all controls handheld. Now, only six surgeons performed all the cases, with some surgeon’s contributing only robotic or laparoscopic data, and clearly each surgeon’s individual posture influences the weight of these results significantly, as does robotic experience, which may influence posture over time.

### 2.2 Senhance®

The origins of the Senhance system are in the Italian TELELAP ALF-X® (SOFAR) robotic platform which was acquired by TransEnterix (USA) in 2015. TransEnterix changed its name to Asensus in 2021 and was bought by Karl Storz in 2024. Senhance is yet another open console modular platform but one of its differentiating features is infrared eye tracking capability which can control the endoscope [18]. Instruments are re-useable, available in 3 mm diameter and compatible with standard laparoscopic ports. This system has an ultrasonic 3 mm dissector available, which got CE approval in 2018 and FDA-approval in 2019. Of note, there was a Class I recall in 2023 for this system because the robotic arm could spin uncontrollably when the teleoperation system was disengaged. No patient harm was reported and after a software update this recall was terminated in 2024.

Spinelli et al. were the first to report on Senhance in colorectal surgery, in an Italian series of 45 patients having major resection for colorectal cancer (66%), diverticular disease (11%), IBD (18%) or endoscopically irresectable polyps (4%) [19]. Median docking time was 11 min and total operative time 256 min. There were no open conversions. Median LOS was 5 days. Major complications occurred in two patients (4%) and there were no post-operative deaths.

In 2019, Darwich and colleagues described their method and early experience of sigmoid diverticular resection [20]. They describe optimal arm positions for different phases (e.g. splenic flexure mobilization, lateral colonic mobilization) using four robotic ports. Of twelve procedures, there were no open conversions. Mean docking time was 10 min, console time 149 min and operative time 219 min. Mean LOS was 9 days. There was one major complication (occult colonic perforation at splenic flexure) and no post-operative mortality. The authors pointed out that the cost per case is likely to be much less than for the DaVinci Xi, at an average of 800 euros per case.

A Taiwanese series of 46 patients (85% with CRC; 4% diverticular disease; 7% irresectable polyps; 4% rectal prolapse) was published in 2021 [21]. Median docking time was 25 min, console time 112 min and total operative time 283 min. Four patients (9%) had a major complication, including one post-operative death due to myocardial infarction. A univariate analysis suggested that major complications were associated with older age, higher ASA grade and right sided resection. The docking time seem unusually protracted compared to similar studies and although the authors do not comment, it is possible that this reflects the surgeon and theatre team being early in their learning curve. In addition, the authors reported that the in-built sensor (force feedback mechanism) exceeded maximum force when lifting the rectum anteriorly with robotic instruments and although the description is vague, it seems that these procedures were completed laparoscopically or at least in a hybrid manner. Unusually, there is no mention of conversion rate to laparoscopic or open surgery. Of note, the authors used straight stick robotic instruments rather than wristed instruments for this series of procedures. Asensus makes non-articulating instruments for surgeons who want to utilize the optics and stability of the robotic platform, with the familiarity of a laparoscopic straight stick.

In 2022, a Lithuanian series reported on 57 patients who had colorectal resection (84% cancer, 16% benign) [22]. Mean operative time was 194 min. There were two open conversions (3%). Mean LOS was 8 days. Four patients had a major complication (6%) but there were no post-operative deaths. Thirty-two percent of patients had rectal resection and the authors commented that they benefited from the articulating Senhance instruments for pelvic dissection. For the four patients who had trans-anal TME, the perineal dissection was done in an open fashion.

In Japan, three-arm robotic resection has been done using a single-incision laparoscopic surgery (SILS) port plus two robotic Senhance ports (SILS+2-S) [23]. The umbilical SILS port is used for the endoscope and an emergency assistant port. The two robotic ports are positioned cranial and caudal to this. Eight patients had resection for colon cancer (right hemicolectomy, sigmoid colectomy, anterior resection). Mean console time was 139 min and operative time 229 min. There were no open conversions. Mean LOS was 7 days. There were no major complications. The authors describe that this technique is feasible for procedures that generally progress in one direction such as right colectomy and sigmoid resection (caudal to cranial), rather than low anterior resection for example. The same group incorporated these data into a larger series of 55 patients having colorectal cancer resections, with and without the SILS+2-S port configuration [24]. There were 27 right-sided and 28 left-sided resections. Median docking time was 8 min, console time 126 min and operative time 260 min. Median LOS was 7 days. There were no major complications. The authors felt that Senhance was an extension of laparoscopic surgery, for which the learning curve was short, but it lacked the versatility of DaVinci X/Xi in more complex procedures such as rectal resection. The authors hint that this was due to their choice of straight stick instruments.

Focusing on rectal cancer, the same team reported outcomes of 16 patients having low anterior resection for T1-4 tumors [25]. They only utilized three robotic arms due to the limited volume of the pelvis, despite the modest median BMI of 20 kg/m^2^, and re-docked for the pelvic phase of the procedure. Median operative time was 309 min. There were no open conversions but in five cases they reverted to laparoscopic instruments. There were no major complications. Median LOS was 8 days. There were two R2 resections with the explanation of distant synchronous tumors. Five patients (>30%) had recurrence (one local recurrence) during a median 36 month follow up period. With these data, the authors acknowledged the limitations of Senhance in rectal cancer surgery.

McKechnie and colleagues have meta-analyzed data from some of these series, combining data from 223 patients having colorectal resection for cancer (81%), diverticular disease (9%), IBD (4%) and endoscopically irresectable polyps (5%) [26]. Overall mean docking time was 11 min, console time 141 min and operative time 230 min. Open conversion rate was 1.3%. Mean LOS was 7 days. Major complications occurred in 5.4% of patients and there was one post-operative death (0.4%). The authors have reasoned that this system is comparable to DaVinci in terms of operative times and short-term outcomes for major colorectal resection. However, only 17% of patients had rectal resection.

### 2.3 HUGOTM

HUGO is another open console modular system, designed and marketed by Medtronic (USA). It has pistol grip hand controls and foot pedals to control the endoscope and switch arms. It has an associated Touch Surgery DS1 computer system which records and uploads procedure recordings to the cloud and artificial intelligence capability to blur out faces, remove patient identifiable information and segment videos into procedural steps. There are reports of its haptic feedback capability but in the colorectal studies mentioned below, the authors do not highlight or discuss this, and the current manufacturer website does not draw attention to this, suggesting that this technology is limited or not fully functional. Furthermore, HUGO currently lacks integrated robotic energy device and stapler but these are in the pipeline and prototypes have been tested [27]. Despite being developed in the USA, it received European certification (2021) before FDA approval (2025), and FDA approval is for urological surgery only.

In March 2023, Bianchi et al. published the first report of HUGO in colorectal surgery, with a series of three patients from a single Italian center [28]. Two patients had right colectomy and one had left colectomy, all for cancer. Docking time ranged from 6-10 min and operative time 255-264 min. There were no open conversions. LOS was 4-6 days. There were no major complications.

Shortly after this, another Italian group reported their early experience [29]. Of 17 patients having a range of abdominal procedures, four had right-sided colonic resection and one had left sided resection. There were no open conversions, major complications or post-operative deaths. The authors highlight that, like other modular systems, docking is completely manual and not semiautomated like the DaVinci Xi, which has a targeting function that directs the boom according to the target anatomy. Furthermore, HUGO has longer robotic arms than DaVinci Xi, meaning that they can limit or obstruct the bedside assistant. Finally, the HUGO endoscope requires an 11mm trocar and cannot be hopped into one of the other 8mm robotic ports.

Belyaev and colleagues published a series of 25 patients having colorectal surgery at a single German center [27]. Four had right-sided resections, 19 had left-sided resections and two had reversal of Hartmann’s. Median operative time was 201 min for left colectomy/ sigmoid colectomy, 345 min for low anterior resection, 312 min for right colectomy and 295 min for reversal of Hartmann’s. Mean docking time for all patients was 12 min. Two patients had a presacral hematoma that required percutaneous drainage and one patient had anastomotic leak managed by endoVAC therapy, giving a major complication rate of 12%. There were no post-operative deaths. The authors state their frustration at a lack of integrated energy device, which particularly hampered progress during right colectomy. They describe their technique of the console surgeon displaying the tissue with a right- and left-handed grasper and the bedside assistant using a laparoscopic energy device to divide the tissue. This is borne out in operative times for right colectomy being over 100 min greater than for left or sigmoid colectomy. They go as far as to say that CME is not appropriate for HUGO until a compatible energy device is available. For Davinci users, this will seem unusual as most technical reports for CME describe traction and counter-traction with right- and left-handed retracting instruments and dissection with monopolar scissors or hook [28]. Of note, the same group published a series of 70 patients having abdominal surgery using HUGO, which seems to include the 25 colorectal patients described above as well six additional patients having colorectal surgery (one sigmoid colectomy, one low anterior resection and four right hemicolectomies) [30]. In the additional patients there were no major complications or deaths. The consolidated median operative time went down to 219 min for right colectomy, suggesting that patient selection or operative technique changed significantly in the additional cases. It is also possible that not all the 25 cases in the initial report were included in the consolidated report.

Romero-Marcos et al. presented their first 10 colorectal cases using HUGO at a single Spanish center in 2024 [31]. Two surgeons carried out five right colectomies, three sigmoid colectomies, one high anterior resection and one VMR. Median docking time was 14 min (15 min for right sided and 14 min for left sided procedures) and operative time 185 min (190 min for right sided and 170 min for left sided procedures). LOS ranged from 2-4 days. There were no major complications. The authors acknowledge the relatively lengthy docking time, which may be related to their inexperience. In their technical analysis they report clashing between the endoscope port and arm-4 at the edge of a particular surgical quadrant (e.g. the terminal ileum during right colectomy), which they solved by leaving arm-4 idle or by hopping the dissector from arm-3 to arm-4.

The first rectal cancer resections using HUGO were reported in 2024 by Caputo and colleagues from a single Italian center [32]. Three patients had low anterior resection. The authors used four robotic arms (cadiere, bipolar forceps, endoscope and scissors), utilizing an assistant port for laparoscopic clip application and stapling. They modified their port set up for the final case to avoid having to re-dock for the pelvic phase. Median docking and console times were 12 and 337 min respectively. All resections were pathologically R0. There were no major complications, and median LOS was 8 days.

The largest HUGO colorectal series was published in 2024, reporting on 40 patients who had resectional surgery (15 right-sided and 25 left-sided (including eight rectal resections)) for cancer, diverticular disease and IBD at a single Spanish center [33]. For right and left sided resections, a standard medial to lateral approach was used, with robotic dissection followed by laparoscopic clipping or stapling of the vascular pedicle. Median operative time was 206 min, with median docking time of 7 min. There were no open conversions. Median LOS was 3 days. Major complications occurred in 3/40 (7.5%) but there were no post-operative readmissions or deaths.

Interestingly, the authors thought that the four arm carts took up a significant amount of space and obstructed the assistant at times. However, they highlighted the value of the single open console in terms of demonstrating anatomy and operative steps.

### 2.4 Dexter®

Dexter from Distalmotion in Switzerland is another open console modular system, similar to Versius, which got European certification in 2020 and FDA approval in 2024 (inguinal hernia) and 2025 (hysterectomy). The range of wristed instruments is also similar to Versius, without an integrated energy device or stapler. Unlike Versius, it does have foot pedals for clutching and moving the endoscope. Another key difference is that the surgeon console is draped and kept sterile so the surgeon can switch to bedside operating in a matter of seconds, which is termed ‘on demand robotics’.

The first report of Dexter in colorectal surgery was from Switzerland in 2023 [34]. Of 12 patients, nine had right sided resection (benign and cancer), one had a transverse colectomy (cancer) and two had ventral mesh rectopexy (VMR). For right hemicolectomy, the ileocolic pedicle was divided centrally using the robot (regardless of pathology), followed by laparoscopic lateral mobilization. In the four patients who had intracorporeal ileo-colonic anastomosis, the stapling was done laparoscopically and the common enterotomy was closed robotically. This highlights the ‘on demand’ nature of the Dexter system. In contrast, all steps of the VMR were completed robotically. For right-sided resections, median operative time was 147 min and for VMR this was 167.5 min. There were no open conversions. Median LOS was 7 days for right colectomy and 4.5 days for VMR. One patient had a major complication (anastomotic bleed requiring endoscopic intervention).

Conrad et al. published a series of 56 patients, with 20 patients having colorectal procedures (16 right-sided resections, two left-sided resections, two rectopexies) [35]. Median operative time for right hemicolectomy was 159 min, anterior resection 112 min and rectopexy 150 min, with median docking times of 10 min or less for each type of procedure. There were no open conversions. Three patients (15%) had a major complication, all requiring re-operation. Although this complication rate seems out of proportion to Versius data, the patient numbers are small and it would be unfair to make any strong judgements without larger series. Median operative time was lower in this Dexter study than in the Versius registry data for all colorectal procedures [16, 35]. This may reflect the hybrid approach used by the surgeons in the Dexter study, switching rapidly to a laparoscopic approach for more challenging parts of the procedure such as flexure mobilization [35]. To some, this rationale will seem paradoxical, seeing as the robotic system is designed to overcome challenges faced with laparoscopic surgery, such as splenic flexure mobilization, but it does highlight that switching between robotic and laparoscopic approaches with the Dexter system is efficient.

More recently, the same group published a comparative analysis of 75 patients having right hemicolectomy with complete mesocolic excision (CME) for colonic cancer [36]. Twenty-five procedures were carried out using Dexter, 25 using DaVinci Xi and 25 with traditional laparoscopic surgery, all by a single surgeon, without randomization. A subileal approach was used for all robotic procedures and standard medial to lateral approach for laparoscopic procedures. Median operative time was comparable in the Dexter (165 min) and laparoscopic (153 min) groups and significantly higher in the DaVinci group (191 min). Mean docking time was 6 min for Dexter and 10 min for DaVinci. Intracorporeal anastomosis was conducted in 100%, 96% and 56% of cases in the Dexter, DaVinci and laparoscopic groups. There were no open conversions in the Dexter or DaVinci groups and one (4%) in the laparoscopic group. Median LOS was comparable between groups (four, four and five days respectively). There was one anastomotic leak in each of the robotic groups and two in the laparoscopic group. One patient in the Dexter group died from cardiac arrest due to critical aortic stenosis. Disease free and overall survival was similar between groups, but median follow up was only 17 months and the study was not powered for survival analysis. Although this is the only comparative analysis to date, the fact that one surgeon completed all cases does introduce significant technical bias. It is unclear how generalizable the results are.

### 2.5 DaVinci® SP

The DaVinci SP system is a closed console, single arm system, which can incorporate an endoscope and three additional instrument arms, inserted through a single 2.5 cm cannula. The beauty of this system is that there are no collisions due to the single arm. Each instrument has an ‘elbow’ and ‘wrist’ joint to facilitate triangulation. The instruments include forceps, scissors, needle driver and clip applicator but there is no integrated stapler or energy device. The ‘extended’ range of instruments are longer and have greater wrist articulation. Most of the colorectal data pertaining to the SP comes from South Korea because FDA (2025) and European (2024) approvals have only recently been agreed.

The first report of the SP in colorectal surgery was in 2021 [37]. Five patients had right colectomy for cancer. The specimen was delivered through the single port and the anastomosis created extracorporeally. Median docking time was 4 min, console time 105 min and operative time 160 min. There were no conversions to multiport or open surgery and there were no major complications. This study demonstrated feasibility and safety in major resection.

Lim and colleagues then published a comparative analysis of patients have single port robotic (DaVinci SP) or laparoscopic right hemicolectomy for cancer [38]. Forty-one patients had robotic surgery and 100 had laparoscopic. There were no open conversions in the robotic group and two in the laparoscopic group. Operative time was significantly higher in the robotic group (182 vs. 130 min). There were no major complications in either group. From a pathological perspective, lymph node harvest did not differ significantly between groups.

Kim et al compared DaVinci SP and traditional multiport laparoscopic surgery for right- and left-sided colonic cancer [39]. Forty-three patients had resection using SP and 97 laparoscopically. Mean operative times were 233 and 204 min respectively. Open conversion rates were 0 and 2% respectively. Patients in the SP group had faster time to gut function and less opiate use. Major complications were only recorded in the laparoscopic group (zero vs 4.1%). All resections were R0 in both groups and lymph node harvest did not differ.

Another Korean study compared DaVinci SP and Xi for colonic cancer resection [40]. Fifty-three patients, across two centers, were assigned to each group by propensity matching. Median operative times were 150 and 153 min respectively. There was one open conversion in the SP group (2%) and none in the Xi group. There were no differences in overall complication rate, however, patients in the SP group had less post-operative pain and a shorter LOS (five vs. six days). This was attributed this to the shorter incision length (5.0 vs. 9.4 cm). The authors conceded that the SP system may not be suitable for rectal resection as the distal transection must be done using a laparoscopic stapler. Equally, they felt less confident without an advanced energy device in the pelvis.

Kim et al. published a series of 50 patients, including 16 having rectal resection [41]. Thirty-nine patients (78%) had cancer resections and the remainder benign. There were 15 right and 35 left-sided resections. Median docking time was 14 min, console time 200 min and operative time 262 min. Median LOS was 7 days. There were no major complications. This group showed that low anterior resection was feasible with SP, acknowledging that rectal transection had to be done with laparoscopic stapler through an additional 12mm port.

A phase II trial of SP from the USA was published by Marks et al in 2023 [42]. Seventy percent of patients had colonic or rectal resection and 30% had trans anal local excision. Of 93 patients having major colorectal resection or continuity surgery, 40% had rectal cancer, 28% diverticular disease, 14% colon cancer, 9% IBD and 4% irresectable polyps. There were eight (9%) right sided procedures and 85 (91%) left sided procedures (including two Hartmann’s reversals and two resection rectopexies). Thirty patients (32%) had a combined trans-abdominal/ trans-anal TME (rTATA), where the robotic port was placed trans-anally for the rectal dissection and the abdominal component was done laparoscopically. Mean operative time was 357 min. Stabilization of docking time occurred after 34 cases. There were no open conversions but conversion to traditional laparoscopic surgery was made in two cases. The major complication rate was 2% (one pelvic collection and one anastomotic leak). Of the forty-three patients with cancer in this group, none had positive resection margins. At a median follow up of 20 months, there were no local recurrences and one distant recurrence (1%). Despite these promising data, the authors reiterate that a lack of robotic suction, energy device and stapler makes trans-abdominal pelvic surgery challenging and they have decided to limit use of the SP to trans-anal local excision and rTATA.

A meta-analysis of these studies and other smaller series of the SP in major colorectal surgery was published in 2025, including 396 patients [43]. Three hundred and forty patients (86%) had cancer resection. There were 123 (31%) right sided procedures, the rest were left-sided (including two rectopexies and one Hartmann’s reversal). Median docking time was 6 min, console time 105 min and operative time 186 min. Median LOD was 8 days. Although complications were not categorized by severity, anastomotic leak occurred in five patients (1%). This seems very low considering that 70% of patients had left sided procedures, even if we consider that not all these had primary anastomoses. No post-operative deaths were recorded within 30 days. In terms of oncological outcomes, the CRM was positive in 2/280 (<1%), where reported. This is very low, but it does include colonic as well as rectal resections. Median lymph node harvest was 25.

In another meta-analysis of 465 patients (83% with CRC), docking time ranged from 12-20 min and median operative time was 225 min [44]. LOS was 3-11 days. Anastomotic leak rate was 4%. Ninety-five percent of patients had an R0 resection and lymph node harvest ranged from 15-28. Included in this review were a very small number of local trans-anal excisions (1%). Combined conversion rates were not documented.

### 2.6 Other notable systems

The SSI Mantra (SS Innovations, India) is an open console, modular offering from India, again lacking integrated energy device and stapler. It has gained popularity for its remote telesurgery applications [45]. Initial data from 32 patients having rectal resection (88% low anterior resection; 12% APR), showed a mean docking time of 21 min, console time 210 min and operative time 270 min [46]. There were no open conversions. LOS was 5 days on average. All resections were R0 with a mean of 26 lymph nodes. Complications were not specifically reported. A subsequent consolidated series of 92 patients from the same group showed an improved mean total operative time of 220 min, an open conversion rate of 2%, 99% R0 resection rate, 2% major complication rate and a mean LOS of 5 days [47].

The Japanese Hinotori system by Medicaroid was approved for use in Japan in 2020 for Urology and 2022 for gastrointestinal and gynecological surgery. This has a closed console and four arms emanating from single Davinci-style tower. Each arm has eight degrees of freedom, more than the typical seven seen in other systems, which may help to improve flexibility and reduce arm collisions. Unlike DaVinci’s multiport systems there is no integrated energy device. Katsuno and colleagues proposed a double bipolar method, using a bipolar forceps in each hand to compensate for this [48]. In a 2:1 propensity matched analysis of DaVinci Xi (108 patients) and Hinotori (54 patients), median console (110 vs. 156 min) and operative (227 vs. 266 min) times were shorter with DaVinci [49]. Median LOS was greater in the DaVinci group (14 vs. 11 days). There were no differences in blood loss, complication rates or oncological parameters. The authors suggested that efficiency was limited with Hinotori because the assistant had to laparoscopically transect the bowel or seal blood vessels. They felt that advancements in this area would improve applicability and uptake.

The MIRA surgical system (Virtual Incision, USA) is a miniaturized device inserted through a single hand-assist port, with the two instrument arms (typically bipolar forceps and monopolar scissors) opening inside the peritoneal cavity. As opposed to mainframe robotics, here, the arms are individually motorized. The system has a standard open console and has been used as part of a multicenter trial in the USA [50]. In this study, dissection was carried out robotically and resection and anastomosis extracorporeally. Data on thirty patients (15 having right and 15 left colectomy) showed median console time of 70 min and operative time 146 min. There were no open conversions. Median LOS was 2 days. The major complication rate was 3%. This system is portable and can be re-sterilized 15 times but only two robotic instruments can be used at the same time, necessitating a hybrid approach.

Two novel closed console fixed arm systems which use the DaVinci Xi as their blueprint have emerged from China and Korea. Hong Kong-based Cornerstone Robotics have developed the Sentire system, which is currently being used in the UK for colorectal surgery as part of a trial [51]. The Korean Revo-i system has been used in over 100 colorectal procedures according to the manufacturer, but data is yet to be published [52]. The Avatera system (Avateramedical, Germany) is another more compact closed console fixed arm system which has been used in preclinical trials, but real-world colorectal data is awaited [53].

## 3 Discussion

In recent years, several developers have tried to capitalize on the lapse of patents and perceived flaws of Intuitive’s multiport systems. In general, there has been an influx of open console, modular systems to address better intraoperative communication and portability (Table 1). It would be unfair to say that these systems are all the same because certain features such as eye tracking and force feedback are unique but the blueprint for many is the same. Clearly, the DaVinci SP is distinct, and although single port colorectal resection is possible, the feeling is that multiport systems offer greater efficiency.

Generally, the perception is that these systems have a reduced initial outlay and lower operating costs compared to DaVinci systems. Our understanding is that capital expenditure is likely to be within two-fold for most systems but contrary to expectation, a recent report suggest that for certain procedures such as prostatectomy, overall cost per procedure (equipment, consumables, theatre costs) may actually be less for DaVinci multiport than other systems [54]. The difficulty with comparing costs is that upfront costs and service contracts are individualized and depend on number of consoles, number of systems in the institution and volume of surgery. Our feeling is that the cost difference between DaVinci and others may not be as much as we might think.

Data for novel systems largely hinges on case series, which seem to have demonstrated safety and efficacy, with operative times, conversion rates, LOS and complication profiles in line with DaVinci X/Xi. However, there are very few comparative studies and no randomized trials to directly assess their performance against traditional laparoscopic surgery or Intuitive’s multiport offerings. That said, randomized trials in surgery remain challenging to conduct due to the technical learning curve, variation in technique and inherent problems with blinding [55].

Unfortunately, a recurrent theme reported in numerous series is the lack of integrated articulating energy devices and staplers for novel systems, the development of which have been limited by remaining active patents and cost of production. However, Senhance has 3 mm ultrasonic shears, the Versius Plus system offers ultrasonic shears and HUGO’s energy device is in the late preclinical stage of testing. Undoubtedly, these advancements will make these systems more efficient and appealing but the additional cost is unclear.

Another consideration with novel systems is that there is less experience to guide the set up and operative technique. Equally, there are fewer surgeon proctors. This combination means that the learning curve may be longer and initial operative times may be higher than what one might see on the DaVinci multiport systems, where more than a decade of experience is available for reference in publications and video vignettes. Procedures may also be less standardized because fewer surgeons are using each new system. This will almost certainly have an impact on training future surgeons.

Unlike DaVinci, not all novel systems have dual console capability. Versius and HUGO offer a dual console set up but in general, open console systems are often used in a single console format. Training using a single console is often less efficient for the procedure itself because the proctor and trainee may have to swap positions throughout the case, and the learning experience is also impacted. Work by Liu et al. using the SimNow® platform has shown that trainee efficiency and accuracy is improved, and cognitive burden is reduced when learning on a dual console system [56].

Robotics has enabled and popularized telesurgery, with the initial concept developed for remote surgery on space crew [57]. Recent trials have shown non-inferiority of telesurgery compared to local surgery in urological procedures [58]. This may allow technical expertise to be delivered to remote areas but it does rely on a robotic system to be installed at the end-user location. On the other hand, telementoring, offered by most of the new players (Versius, HUGO, Senhance, Dexter), allows for remote proctoring and troubleshooting without the need for an additional console, and has been used extensively across the world.

## 4 Conclusion

For now, what we can say, is that novel robotic systems are safe and effective in colorectal resection for benign and malignant disease. How they stand up directly to Davinci’s multiport systems has not been shown clearly, but in single center series and meta-analyses of these series, the numbers are comparable. The feeling is that these systems may prove to be a viable cost-effective option with the drawback of premium usability, until the whole gamut of robotic instruments is fully integrated. Undoubtedly, newer medical robotics companies will have to ensure that engineering, software, artificial intelligence integration, and training capability are fully funded through research and development, in order to flourish in this market.

## Figures and Tables

**Table 1:** A summary of alternative robotic systems in colorectal surgery

System	Console	Patient cart	Initial cost ($)*	FDA/ CE approval	Features	Relevant colorectal studies
**Versius® (CMR, UK)**	Open	Modular (5-arm)	1.4-1.9 million	FDA 2024 (chole). CE 2019 (adult laparoscopic).	Up to five arms. No foot pedals.	[10–14, 16, 17]
**Senhance (Asensus, USA)**	Open	Modular (4-arm)	1.2-1.5 million	FDA 2017 (Colo/ Gynae); 2024 (Gen/ Paed/ Urol). CE 2014 (abdomen/ pelvis).	Eye tracking. Force feedback. Ultrasonic shears. 3mm instruments.	[19, 21, 22, 24, 25]
**HUGO™(Medtronic, USA)**	Open	Modular (4-arm)	1.2-1.5 million	FDA 2025 (Urology). CE 2021 (Urol/ Gynae); 2022 (Gen).	Touch Surgery™ DS1 AI interface	[27, 29, 30, 33]
**Dexter®, (Distalmotion, Switzerland)**	Open	Modular (4-arm)	0.5-1 million	FDA 2024 (inguinal hernia); 2025 (chole/ Gynae). CE 2020 (Gen/ Gynae/ Urol).	‘On demand’ robotics.	[34–36]
**DaVinci® SP** **(Intuitive, USA)**	Closed	Single arm (3 instruments)	2.5 million	FDA 2019 (Urol/ trans-oral); 2025 (trans-anal); 2025 (Gen). CE 2025 (Urol/ ENT/ trans-anal/ thoracic).	Single arm with endoscope and three instruments.	[37–44]
**SSI Mantra (SS Innovations, India)**	Open	Modular (4-arm)	1-1.5 million	Awaited		[46, 47]
**Hinotori™ (Medicaroid, Japan)**	Closed	Single pivot tower	1.5 million	Awaited	Arms with eight degrees of freedom.	[49]
**MIRA® (Virtual** **Incision, USA)**	Open	Single arm (2 instruments)	Undisclosed	Awaited	Miniaturized. Motorized arms.	[50]
**Sentire® (Cornerstone Robotics, China)**	Closed	Single pivot tower	Undisclosed	Awaited		Awaited
**Revo-i (Surgical Robotics Technology, Korea)**	Closed	Single pivot tower	Undisclosed	Awaited		Awaited

* Capital expenditure varies according to individual contract and approximate costs are presented. Chole – cholecystectomy; Gynae – gynaecology; Urol – urology; Gen – General surgery; Paed – paediatric surgery.
